# Chemical Composition of Essential Oils from Different Parts of *Zingiber* *kerrii* Craib and Their Antibacterial, Antioxidant, and Tyrosinase Inhibitory Activities

**DOI:** 10.3390/biom10020228

**Published:** 2020-02-04

**Authors:** Aknarin Pintatum, Surat Laphookhieo, Emilie Logie, Wim Vanden Berghe, Wisanu Maneerat

**Affiliations:** 1 Center of Chemical Innovation for Sustainability (CIS) and School of Science, Mae Fah Luang University, Chiang Rai 57100, Thailand; p.aknarin@gmail.com (A.P.); surat.lap@mfu.ac.th (S.L.); 2 Lab Protein Chemistry, Proteomics & Epigenetic Signalling (PPES), Department Biomedical Sciences, University of Antwerp, 2610 Wilrijk, Belgium; emilie.logie@uantwerpen.be (E.L.); wimk.vandenberghe@gmail.com (W.V.B.)

**Keywords:** *Zingiber kerrii*, chemical composition, biological activity, anti-tyrosinase, cytotoxicity

## Abstract

The essential oils of the fresh rhizomes; flowers; and leaves of *Zingiber kerrii* Craib were investigated using different extraction techniques; including solid-phase microextraction (SPME), hydrodistillation (HD), and organic solvent (OS), and characterized by gas chromatography–mass spectrometry (GC–MS). A total of 37 SPME; 19 HD; and 36 OS compounds were identified from the rhizome extract of *Z. kerrii;* with the major components being α-pinene; β-pinene; and terpinen-4-ol; respectively. From the flower extract; 16 SPME; 2 HD; and 10 OS compounds were identified; (*E*)-caryophyllene was found as a major compound by these techniques. The leaf extract exhibited 20 SPME; 13 HD; and 14 OS compounds; with α-pinene; (*E*)-caryophyllene; and *n*-hexadecanoic acid being the major compounds; respectively. The rhizome extract showed tyrosinase inhibitory activity of 71.60% and a total phenolic content of 22.4 mg gallic acid/g. The IC_50_ values of the 2,2-diphenyl-1-picrylhydrazyl (DPPH) and 2,2′-azino-bis(3-ethylbenzothiazoline-6-sulfonic acid) diammonium salt (ABTS) assays were 25.2 µg/mL and 153.6 µg/mL; respectively; and the ferric ion reducing antioxidant power (FRAP) assay value was 318.5 µM ascorbic acid equivalent (AAE)/g extract. The rhizome extract showed weak antibacterial activity. This extract showed no adverse toxicity in human keratinocyte (HaCaT) cell lines at concentrations below 200 µg/mL.

## 1. Introduction

Zingiberaceae is a large family containing approximately 50 genera and over 1200 species distributed throughout the tropical countries [1,2,3]. This family is known to produce complex mixtures of volatile substances. The traditional essential oils from these plant species are obtained from different extractions, including hydrodistillation, steam distillation, and solvent extraction [4,5]. Organic solvent (OS) extraction is suitable for delicate raw materials, including flowers. Hydrodistillation (HD) is a common technique for the extraction of essential oils from rhizomes and aerial parts [4]. In addition, solid-phase microextraction (SPME) is a rapid, simple, inexpensive, and solvent-free technique. SPME requires little raw material and obtains only highly volatile compounds [6,7], whereas HD and OS extraction may lose some highly volatile compounds during solvent removal via evaporation and recover non-volatile compounds via OS extraction [5,8,9]. Therefore, the content and the chemical composition of an essential oil may vary according to the extraction method.

Recently, monoterpenes, oxygenated monoterpenes, sesquiterpenes, oxygenated sesquiterpenes, phenylpropanoids, and miscellaneous compounds were identified from the essential oils of some *Zingiber* species [1,9,10,11]. Thirty constituents comprising 99.99% of the total material were identified in fresh rhizome essential oils of *Z. zerumber* using gas chromatography-mass spectrometry (GC-MS). The major constituents included zerumbone (74.82%), humulene (6.02%), and *β*-copaen-4*α*-ol (4.32%) [12]. The chemical composition of the essential oils from the leaves and the rhizomes of *Z. cassumunar* included 64 and 32 components, accounting for 94.60% and 98.56% of the oils, respectively. Sabinene (14.99%) and *β*-pinene (14.32%) were identified as the major components in the leaves, and triquinacene,1,4-bis(methoxy) (26.47%), (*Z*)-ocimene (21.97%), and terpinen-4-ol (18.45%) were reported as the major components of *Z. cassumunar* rhizomes [13]. Moreover, 37 and 34 chemical components were identified in the rhizome essential oils of *Z. officinale* and *Z. amaricans*. (*E*)-Citral (20.98%) and zerumbone (40.70%) were exhibited as the main compounds in the essential oils [14].

Previous reports showed that many species of the Zingiberaceae family possessed antioxidant properties, such as *Z. officinale* [15,16], *Z. zerumbet* [12,17,18], and *Curcuma longa* [15]. The rhizomes of *Z. officinale* are used in traditional medicine for stomach-aches, gastric ulcers, and flatulence in Thailand, and *Z. zerubet* rhizomes are used as traditional medicines for the treatment of swelling, coughs, colds, stomach-aches, and skin diseases. The rhizomes of *Z. zerumbet* contain the main compound of zerumbone, which showed antioxidant, antimalarial, and cytotoxic activities [12,18]. *Z. cassumunar* rhizomes are used as traditional medicine in Thailand and Indonesia; these were shown to possess strong antioxidant and anti-inflammatory activities [19,20]. Moreover, the rhizomes of *Z. montanum* are used for the treatment of various diseases in Thailand [21]. The major constituents in rhizome essential oils include terpinen-4-ol and (*E*)-1(3, 4-dimethylphenyl) butadiene, which were shown to demonstrate high antioxidant, anti-inflammatory, and anti-allergic activities. In addition, the new complex curcuminoids isolated from the rhizomes of *Z. montanum* exhibited stronger antioxidant activities than curcumin [21,22].

A great deal of research has been conducted on the chemical composition and the biological activities of *Zingiber* species; however, *Zingiber kerrii* Craib has not yet been studied. Therefore, this is the first study to investigate the composition of the essential oils from *Z. kerrii* and their antibacterial, antioxidant, cytotoxic, and tyrosinase inhibitory activities.

## 2. Materials and Methods

### 2.1. Plant Material

Fresh flowers, leaves, and rhizomes of *Z. kerrii* were collected from the Doi Tung Development Project, Chiang Rai province, Northern Thailand (N: 20.3261°, E: 99.8275°) at an altitude of approximately 50 m during the rainy season in September 2015. Plant authentication was verified by Mr. Martin Van de Bult, and a voucher specimen (MFU-NPR0197) was deposited at the Natural Products Research Laboratory of Mae Fah Luang University.

### 2.2. Chemicals

Gallic acid, L-ascorbic acid, kojic acid, ferric chloride, 2,2-diphenyl-1-picrylhydrazyl (DPPH), 2,2′-azino-bis(3-ethylbenzothiazoline-6-sulfonic acid) diammonium salt ABTS, 2,4,6-tris(2-pyridyl)-*s*-triazine (TPTZ), C_8_–C_20_ *n*-alkanes standard solution, tyrosinase from mushroom, 3,4-dihydroxy-l-phenylalanine (L-DOPA), 3-[4,5-dimethylthiazol-2-yl]-2,5-diphenyl tetrazolium bromide (MTT), sodium dodecyl sulfate (SDS), and dimethyl sulfoxide (DMSO) were purchased from Sigma-Aldrich (St. Louis, MO, USA). Folin–Ciocalteu’s phenol reagent was obtained from Merck KGaA (Darmstadt, Germany). Mueller-Hinton broth was obtained from HiMedia Laboratories (Mumbai, India). Vancomycin hydrochloride was obtained from the EDQM Council of Europe (Strasbourg, France). Gentamycin sulfate and ampicillin sodium salt were obtained from Bio Basic Canada (Markham, ON, Canada).

### 2.3. Essential Oil Extraction

#### 2.3.1. Solid-Phase Microextraction (SPME)

An SPME fiber coated with 50/30 µm divinylbenzene-carboxen-polydimethylsiloxane (DVB/CAR/PDMS) was used to extract the volatile components from all parts of fresh *Z. kerrii*. The SPME fiber was purchased from Supelco (Bellefonte, PA, USA). Fresh samples (50 g) were transferred to a 250 mL glass septum bottle, then kept in a water bath at 45 °C for 30 min. For each extraction, the SPME fiber was preconditioned for 30 min at 220 °C in the injection port of GC–MS. The fiber was then exposed to the headspace for 30 min. The thermal desorption of constituents was carried out at 250 °C for 5 min.

#### 2.3.2. Hydrodistillation Extraction (HD)

All fresh parts of *Z. kerrii* (100 g) were extracted via hydrodistillation (separately) for 6 h [23] using a Clevenger-type apparatus. The obtained essential oils were dried over anhydrous sodium sulphate. The essential oils were kept in a sealed vial and stored at 4 °C for further studies.

#### 2.3.3. Organic Solvent Extraction (OS)

Soluble compounds from all of the fresh *Z. kerrii* parts were extracted using *n*-hexane as a solvent. A total of 50 g of each sample was weighed and suspended in 300 mL of solvent, then shaken for 6 h in an electronic shaker at room temperature. Whatman No. 1 filter paper was used for filtering. The solvent was removed via reduced pressure with rotary evaporator at 40 °C, then stored at 4 °C for further studies.

### 2.4. Rhizome Extraction

Dried rhizomes (1 kg) were extracted three times with ethyl acetate (EtOAc) for 72 h at room temperature. The resulting mixtures were filtrated, then the solvent was removed at 40 °C using a vacuum to produce the EtOAc extract (7.67 g), which was stored at 4 °C for further studies.

### 2.5. Gas Chromatography–Mass Spectrometry (GC–MS) Analysis

The volatile components of essential oils were performed by GC–MS using the Hewlett Packard model HP6890 gas chromatograph (Agilent Technologies, Santa Clara, CA, USA) with an HP model 5973 mass-selective detector. The analyses were carried out using a capillary column (30 m × 0.25 mm i.d., film thickness 0.25 µm; Agilent Technologies) of HP-5ms (5% phenylpolymethylsiloxane). The temperature program was set at 60 °C and increased to 220 °C at a rate of 3 °C/min. The temperatures of the injector and the detector were set at 250 °C and 280 °C, respectively. The carrier gas was purified helium (99.99%) at a flow rate of 1 mL/min in split mode 1:70 with an injection volume of 1 μL, which was injected 3 times. For electron ionization, mass spectra were used with an ionization energy of 70 eV and ionization voltages over the range of *m*/*z* 29–300. The electron multiplier voltage was 1150 V. The ion source and the quadrupole temperatures were set to 230 °C and 150 °C, respectively. The volatile components were identified by comparing their Kovát retention indices relative to the C_8_–C_20_ *n*-alkanes standard and comparing the mass spectra of individual components with the reference mass spectra via Wiley and National Institute of Standards and Technology (NIST) database matching. The relative concentrations of the volatile compounds were investigated using a percent relative peak area, as shown in Table 1.

### 2.6. Total Phenolic Content Assay

The total phenolic content was determined with the Folin–Ciocalteu assay. The Folin–Ciocalteu reagent was diluted 10-fold with Milli-Q water prior to use [24]. The extracts were prepared at a concentration of 1 mg/mL in ethanol. One hundred microliters of sample was added to 750 µL of the Folin–Ciocalteu reagent and mixed well. The mixture was incubated at room temperature for 5 min, then 750 µL of 6% (*w*/*v*) sodium carbonate was added to the mixture and incubated for 90 min at the same conditions. The absorbance of the mixture was monitored at 750 nm using a UV-Vis Genesys 30 Visible spectrophotometer (Thermo Fisher Scientific, Fitchburg, WI, USA). Gallic acid (5, 10, 25, 50, and 100 µg/mL) was used as a positive control to generate a standard calibration curve. The total phenolic content was expressed as gallic acid equivalents in grams per 100 g extract (GAE/100 g).

### 2.7. DPPH Free Radical Scavenging Assay

One hundred microliters of sample at serially diluted concentrations (5, 10, 25, 50, and 100 µg/mL in methanol) was added to 100 µL (6 × 10^−5^ M) of DPPH methanolic solution, mixed well, and incubated in the dark at room temperature for 30 min. The absorbance of the reaction solution was measured at 517 nm using the microplate reader (Biochrom Asys UVM 340 Microplate Reader, Biochrom, Cambridge, UK). Ascorbic acid at serially diluted concentrations (0.5, 1, 2, 4, and 8 µg/mL in methanol) was used as the positive control. The DPPH radical scavenging activity was expressed as the inhibitory concentration at 50% (IC_50_), which was calculated in comparison with the ascorbic acid [25].

### 2.8. ABTS Radical Cation Scavenging Assay

The working solution of ABTS radical cation (ABTS^●+^) was prepared by reacting 7 mM of ABTS with 2.45 mM of potassium persulfate and allowing the mixture to stand in the dark at room temperature for 16 h before use. Prior to the assay, the working solution of ABTS^●+^ was diluted with ethanol to an absorbance of 0.70 ± 0.05 at 734 nm to give the ABTS^●+^ solution. Twenty microliters of serially diluted sample (50, 100, 150, 200, and 300 µg/mL) was mixed with 180 µL of ABTS^●+^ solution. The reaction of mixture was allowed to stand in the dark at room temperature for 5 min, then the absorbance of the reaction solution was measured at 734 nm. Serially diluted concentrations of ascorbic acid (1.5, 3, 6, 12, and 25 µg/mL) were used as the positive controls. The ABTS radical cation scavenging activity was expressed as the inhibitory concentration at 50% (IC_50_), which was calculated in comparison with ascorbic acid [26].

### 2.9. Ferric Reducing Antioxidant Power (FRAP) Assay

The working solution of FRAP was prepared by mixing 300 mM of sodium acetate buffer at a pH of 3.6 with 10 mM of TPTZ (solution in 40 mM of HCl) and 20 mM of ferric chloride solution in the proportion 10:1:1 (*v*/*v*). The FRAP reagent was prepared fresh daily and warmed at 37 °C in a water bath for 15 min prior to use. Fifty microliters of sample was added to 1.5 mL of FRAP reagent and incubated in the dark at room temperature for 5 min, then the absorbance of the reaction solution at 593 nm was recorded. Ascorbic acid at serially diluted concentrations (50, 100, 200, 300, and 400 µM) was used to generate a calibration curve. The results were expressed as µM ascorbic acid equivalents per gram of extract [27].

### 2.10. Cytotoxicity Assay

Human keratinocyte cells (HaCaT) were seeded at a density of 2 × 10^4^ cells/well into 96-well plates and allowed to adhere overnight. Then, cells were treated with increasing concentrations of plant extracts (12.5–200 μg/mL) for 24 h at 37 °C in 5% CO_2_. MTT solution (5 mg/mL) was added to each well and incubated for another 4 h at 37 °C before dissolving the formazan product in 10% SDS–0.01M HCl. Finally, absorbance was measured at 595 nm after 24 h using a microplate reader (EnVision Xcite 2103-0020 Multilabel Plate Reader, Perkin Elmer, MA, USA). The viability of the cells was reported as the mean ± SD of three independent experiments performed in quadruplicate (Table 3) [28,29,30].

### 2.11. Antibacterial Microdilution Assay

Four Gram-positive bacteria, *Bacillus cereus* TISTR 687, *Staphylococcus epidermidis* TISTR 2141, *Bacillus subtilis* TISTR 1248, and *Staphylococcus aureus* TISTR 746, and four Gram-negative bacteria, *Salmonella typhimurium* TISTR 1470, *Pseudomonas aeruginosa* TISTR 1287, *Escherichia coli* TISTR 527, and *Serratia marcescens* TISTR 1354, were obtained from the Microbiological Resources Centre of the Thailand Institute of Scientific and Technological Research. The minimum inhibitory concentrations (MICs) were determined using Mueller-Hinton broth microdilution with serially diluted (two-fold) plant extracts using 96-well microtiter plates. Vancomycin, gentamycin, and ampicillin were used as the positive controls, while DMSO was used as the negative control (Table 4) [31].

### 2.12. Inhibition of Tyrosinase Assay

The tyrosinase inhibition activity was determined using a slightly modified dopachrome method with L-DOPA as the substrate [32,33]. Briefly, 50% DMSO was used to dissolve plant extracts at a concentration of 10 mg/mL. A 40 µL sample was added to 80 µL of 0.1 M phosphate buffer (pH 6.8), then 40 µL of tyrosinase from mushroom, enzyme commission number 1.14.18.1 (48 units/mL), and 40 µL of L-DOPA (2.5 mM) were added and incubated for 30 min at room temperature. Each sample was accompanied by a blank sample containing all of the components without L-DOPA. The absorbance was measured at 490 nm, with kojic acid used as the positive control. Finally, the inhibition of tyrosinase was calculated (Table 2).

### 2.13. Statistical Analysis

The statistical analyses were performed using IBM SPSS Statistics, version 23 (IBM Crop.). The principal component analysis (PCA) was conducted to reveal interrelationships between plant parts and extraction methods based on measured characteristics. A two-way analysis of variance (ANOVA) was used to compare the volatile constituents exhibited by plant parts after various extraction methods.

## 3. Results and Discussion

### 3.1. Essential Oils Composition

The yields of essential oils (*v*/*w*) from HD and OS extracts of fresh rhizomes, flowers, and leaves of *Z. kerrii* were (0.4%, 0.6%), (0.2%, 0.3%), and (0.2%, 0.3%), respectively. Rhizomes represented the highest percentage of essential oil yields using the OS technique. The chemical compositions of rhizomes, flowers, and leaves essential oils are shown in (Table 1). GC–MS analysis of *Z. kerrii* rhizome essential oils showed 37, 19, and 36 different components using the SPME, HD, and OS techniques, respectively, of which 95.5%, 96.9%, and 90.8% were identified. The essential oils were mixtures of six chemical classes including monoterpene hydrocarbons, oxygenated monoterpenes, sesquiterpene hydrocarbons, oxygenated sesquiterpenes, diterpene hydrocarbons, and fatty acid esters. The major components were *α*-pinene (22.1% ± 0.6%, 24.3% ± 1.6%), *β*-pinene (17.2% ± 0.7%, 33.1% ± 2.5%), sabinene (12.3% ± 1.7%, n/a), and (*E*)-*β*-ocimene (8.1% ± 0.3%, 7.8% ± 0.5%) using the SPME and the HD techniques, respectively, whereas the major components of the OS technique included *β*-pinene (7.7% ± 1.1%), (*E*)-*β*-ocimene (4.1% ± 0.8%), terpinen-4-ol (11.5% ± 0.6%), germacrene B (4.2% ± 0.6%), caryophyllene oxide (4.4% ± 0.7%), and *n*-hexadecanoic acid (8.7% ± 0.9%). The flower essential oils showed 16, two, and nine compounds, of which 99.1%, 97.2%, and 97.1% were identified. The main compound was *E*-caryophyllene (58.1% ± 1.3%, 94.8% ± 0.9%, 74.2% ± 1.5%), which was identified via the SPME, the HD, and the OS techniques, respectively. The SPME technique showed *α*-pinene (22.2% ± 1.8%) and *β*-pinene (8.6% ± 1.1%) as dominant compounds, but *n*-hexadecanoic acid (14.9% ± 0.4%) was as a major compound identified by the OS technique. The isolated essential oils of leaves contained 20, 13, and 14 components, which represented 95.4%, 91.8%, and 93.1% of the total oil compositions, respectively. Most of the compounds in the leaves belonged to five chemical classes, which are listed in (Table 1). The major components of the essential oils in the leaves were *α*-pinene (39.7% ± 0.7%), *β*-pinene (6.3% ± 0.5%), and (*E*)-caryophyllene (21.2% ± 0.8%) using the SPME technique, but the HD technique identified *β*-elemene (12.2% ± 1.3%), (*E*)-caryophyllene (24.2% ± 1.6%), and valencene (21.2% ± 1.3%) as the major components. (*E*)-caryophyllene (6.0% ± 0.8%), isodaucene (4.2% ± 0.9%), and *n*-hexadecanoic acid (55.7% ± 1.3%) were identified as the main compounds when the OS technique was used.

The essential oil compositions of *Z. kerrii* (Table 1) were different according to the extract technique used. (*E*)-Caryophyllene was the main compound seen in the flowers as identified by several techniques, with the exception of the SPME technique in the rhizomes. In addition, *α*-pinene and *β*-pinene were found in several parts, except the flowers when the HD and the OS techniques were used. The results revealed that the HD and OS techniques lost some highly volatile compounds during the process of extraction [5,8,9]. The main compounds usually reported from *Z. officinale* include zingiberene, geranial, and other sesquiterpene hydrocarbons [34,35], whereas literature data on the compositions of *Z. zerumbet* leaves reported (*E*)-nerolidol, *α*-pinene, and *β*-pinene as the main compounds [36]. In our results, *α*-pinene, *β*-pinene, and (*E*)-caryophyllene were found to be the major constituents of *Z. kerrii,* which corroborated previous literature reports of *Z. zerumbet* leaves [36]. This work is the first investigation into the chemical compositions of essential oils from several parts of *Z. kerrii* using different techniques.

### 3.2. Statistical Analysis of Z. kerrii Volatile Components

**Figure 1:**
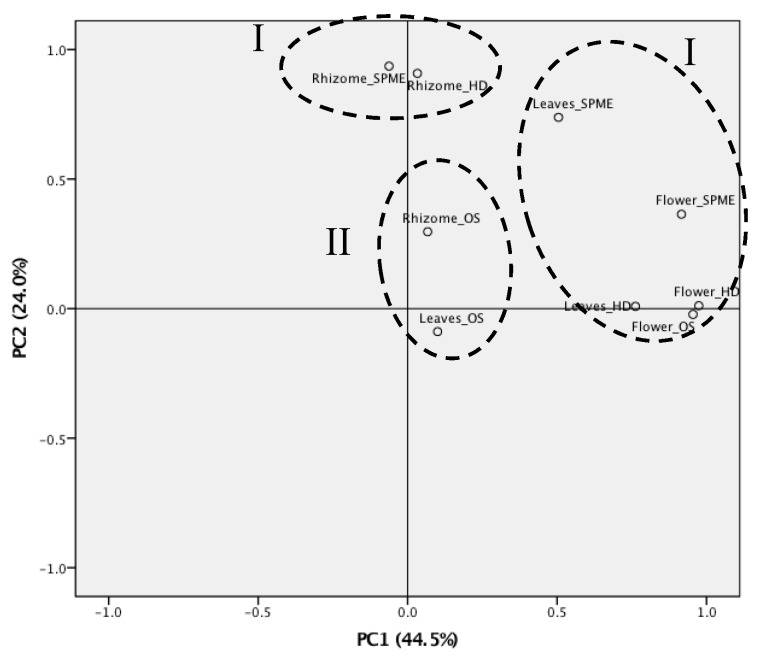
Principal component analysis (PCA) plots of all plant parts and extraction techniques.

**Figure 2:**
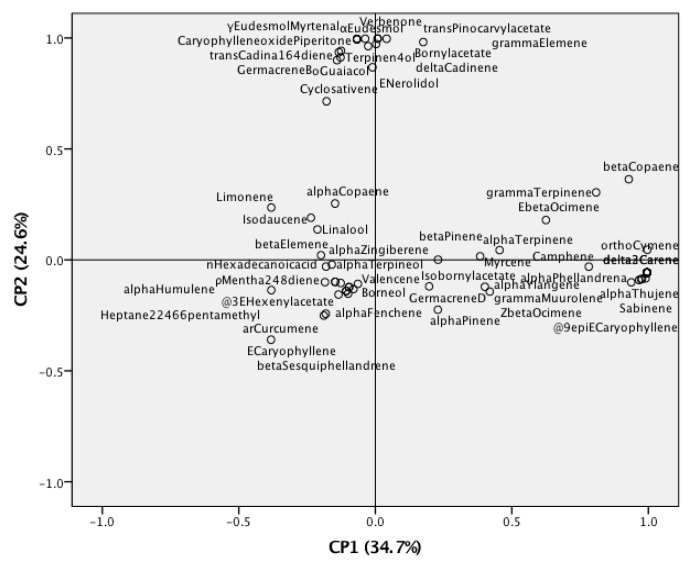
PCA loading plots revealing the compounds present in *Z. kerrii.*

PCA was used to determine the differences in the obtained volatile components of *Z. kerrii* according to the plant part and the extraction technique (Figure 1 and Figure 2). The principal components (PC) were selected according to the highest significance and the explanation of the variation. In the PCA results, all samples were separated into three clusters in the PCA score plot according to the main components (Figure 1). The PCA score plot revealed that the essential oil compositions were associated with the plant part and the extraction method as well as PC1 and PC2. PC1 explained 44.5% of the total volatile component variation and PC2 accounted for 24.0%. The flowers and the leaves exhibited similar major components, but the rhizomes were different. The extraction methods markedly impacted the chemical compositions of essential oils. As shown in Figure 2, the negative axis was highly influenced by *α*-pinene, *β*-pinene, (*E*)-caryophyllene, *β*-elemene, *α*-zingiberene, valencene, (*E*)-*β*-ocimene, terpinen-4-ol, *α*-humulene, linalool, and myrcene, all of which were present in large amounts. This was considered to be the strongest determinant of chemical composition identification.

Two-way ANOVA analysis was performed to define the variables of the chemical composition according to plant part and extraction method, revealing a significant (*p* < 0.05) interaction between the plant part and the extraction method with the volatile components.

### 3.3. Antioxidant Activities and Total Phenolic Content

The total phenolic content and antioxidant activity of the *Z. kerrii* rhizome extract were evaluated using DPPH, ABTS, and FRAP assays. The total phenolic content of the extract was low, at 2.2 ± 0.1 mg GAE/100 g extract. Regarding antioxidant activity, the extract showed IC_50_ values of 143.5 and 169.7 μg/mL using the DPPH and ABTS assays ascorbic acid was used as a positive control, with IC_50_ values of 1.6 ± 0.8 and 5.2 ± 0.8 μg/mL, respectively. The FRAP were also low, at 0.3 ± 0.01 mM AAE/g extract, as shown in Table 2.

Phenolic compounds are secondary metabolites in plants that show strong antioxidant activities [38]. They play important roles in neutralizing free radicals, thereby preventing oxidative damage. Previous research revealed that a relationship between the phenolic content and the antioxidant activity. In this study, the *Z. kerrii* rhizome contained a small amount of phenolic compounds. The chemical compositions of the *Z. kerrii* essential oils showed that terpenoid was a main compound. Similarly, previous studies reported that the phenolic content in leaves was higher than in rhizomes [39], providing the reason why the lowest antioxidant activity was indicated by those assays.

### 3.4. In Vitro Cytotoxicity

To assess cytotoxicity, HaCaT cells were treated with increasing doses of extract, after which cell viability was estimated using the MTT assay. Percentage of cell viability is reported as the mean ± SD of three independent experiments. The cytotoxicity of the extract at a concentration of 200 μg/mL demonstrated a cell viability of 23.1% ± 10.4%, whereas lower concentrations of extract did not inhibit the growth of the cell lines (Table 3). The results revealed that *Z. kerri* extract was non-toxic to the cells in a concentration range of 12.5–100 μg/mL.

### 3.5. Antibacterial Activity

The antibacterial activity of the extract was assayed in vitro using the Mueller-Hinton broth microdilution method against eight types of resistant bacteria. The extract showed the smallest MIC value only to *S. aureus* and *S. typhimurium* in concentrations of 640 μg/mL with less activity toward other bacterial strains in concentrations 1280 μg/mL or more compared with the standard antibiotics vancomycin, gentamycin, and ampicillin, respectively (Table 4).

We found that the antimicrobial activity of *Z. kerrii* extract was different from other *Zingiber* species, such as the *n*-hexane solvent extract of the *Z. officinale* rhizome, which exhibited sensitivity to Gram-positive and Gram-negative bacteria, including *S. epidermidis, S. aureus,* and *E. coli*. It was clear from the results that its extract contained fewer phenolic compounds and oxygenated sesquiterpenes than indicated by previous studies, as these compounds showed antimicrobial potency [40].

### 3.6. Tyrosinase Activity

The extract exhibited moderate tyrosinase inhibitory activity at 22.7 ± 0.8 mg kojic acid equivalence per gram extract (Table 2). In this test, the tyrosinase inhibitory effects of the extract may have depended on the antioxidant properties and the phenolic compounds acting as active sites of tyrosinase and inducing steric or conformational changes, thereby resulting in lower enzymatic activity [41,42,43].

## 4. Conclusions

This is the first report of the chemical profiles of fresh *Z. kerrii* rhizomes, flowers, and leaves, as well as their biological activities. *Z. kerrii* showed moderate activity against bacterial strains and tyrosinase activity. The extract was also non-toxic to human keratinocyte cells at lower than 100 μg/mL concentration; therefore, this is a promising candidate for the development of cosmetic products. Further investigation is required to identify the phenolic compounds of crude rhizome extract.

## Figures and Tables

**Table 1:** Chemical compositions of fresh rhizomes, flowers, and leaves of *Zingiber kerrii.*

Compound	LRI Cal.	LRI Lit.	Rhizomes (%)			Flowers (%)			Leaves (%)			Identification Methods
			SPME	HD	OS	SPME	HD	OS	SPME	HD	OS	
*α*-Thujene	931	930	0.5 ± 0.04	nd	nd	nd	nd	nd	0.3 ± 0.1	nd	nd	MS, LRI, AD
*α*-Pinene	939	939	22.1 ± 0.6	24.3 ± 1.6	3.2 ± 0.3	22.2 ± 1.8	nd	nd	39.7 ± 0.7	1.7 ± 0.1	3.0 ± 1.9	MS, LRI, AD
*α*-Fenchene	953	952	nd	nd	nd	0.5 ± 0.2	nd	nd	nd	nd	nd	MS, LRI, AD
Camphene	954	954	2.6 ± 0.4	1.5 ± 0.3	0.2 ± 0.04	0.4 ± 0.1	nd	nd	nd	nd	nd	MS, LRI, AD
Sabinene	979	975	12.3 ± 1.7	nd	nd	1.3 ± 0.7	nd	nd	1.6 ± 0.5	nd	nd	MS, LRI, AD
*β*-Pinene	983	979	17.2 ± 0.7	33.1 ± 2.5	7.7 ± 1.1	8.6 ± 1.1	nd	nd	6.3 ± 0.5	1.8 ± 0.3	2.6 ± 0.9	MS, LRI, AD
2,2,4,6,6-Pentamethyl heptane	985	-	nd	nd	nd	nd	nd	nd	nd	nd	2.4 ± 1.4	LRI, AD
Myrcene	996	990	1.5 ± 0.5	2.0 ± 0.4	0.4 ± 0.1	0.9 ± 0.2	nd	nd	0.4 ± 0.1	nd	nd	MS, LRI, AD
3-(*E*)-Hexenyl acetate	1002	1002	nd	nd	nd	nd	nd	nd	nd	nd	1.7 ± 1.1	MS, LRI, AD
*δ*-2-Carene	1006	1002	0.5 ± 0.1	nd	nd	nd	nd	nd	nd	nd	nd	MS, LRI, AD
*α*-Phellandrena	1010	1002	0.2 ± 0.1	nd	nd	nd	nd	nd	nd	nd	nd	MS, LRI, AD
*δ*-3-Carene	1015	1011	0.7 ± 0.1	nd	nd	nd	nd	nd	nd	nd	nd	MS, LRI, AD
*α*-Terpinene	1021	1017	0.6 ± 0.1	0.8 ± 0.2	0.1 ± 0.05	nd	nd	nd	nd	nd	nd	MS, LRI, AD
*o*-Cymene	1029	1026	2.7 ± 0.3	nd	nd	nd	nd	nd	nd	nd	nd	MS, LRI, AD
Limonene	1033	1029	nd	4.0 ± 0.6	1.8 ± 0.2	1.7 ± 0.3	nd	nd	1.8 ± 0.2	1.4 ± 0.6	0.5 ± 0.3	MS, LRI, AD
Sylvestrene	1034	1030	3.0 ± 0.3	nd	nd	nd	nd	nd	nd	nd	nd	MS, LRI, AD
(*Z*)-*β*-Ocimene	1041	1037	0.4 ± 0.1	nd	nd	nd	nd	nd	nd	nd	nd	MS, LRI, AD
(*E*)-*β*-Ocimene	1051	1050	8.1 ± 0.3	7.8 ± 0.5	4.1 ± 0.8	1.7 ± 0.1	nd	nd	1.5 ± 0.5	1.2 ± 0.3	nd	MS, LRI, AD
*γ*-Terpinene	1063	1059	1.7 ± 0.3	nd	0.7 ± 0.1	0.9 ± 0.1	nd	nd	nd	nd	nd	MS, LRI, AD
*ρ*-Mentha-2,4(8)-diene	1083	1088	nd	0.8 ± 0.1	nd	nd	nd	nd	nd	nd	nd	MS, LRI, AD
*o*-Guaiacol	1086	1089	nd	nd	1.8 ± 0.3	nd	nd	0.6 ± 0.1	nd	nd	nd	MS, LRI, AD
Terpinolene	1094	1088	0.58 ± 0.18	nd	nd	nd	nd	nd	nd	nd	nd	MS, LRI, AD
Linalool	1106	1096	nd	nd	1.9 ± 0.2	nd	nd	1.1 ± 0.1	nd	4.0 ± 0.5	3.3 ± 0.4	MS, LRI, AD
allo-Ocimene	1133	1132	0.6 ± 0.2	nd	nd	nd	nd	nd	nd	nd	nd	MS, LRI, AD
Borneol	1170	1169	nd	0.8 ± 0.2	nd	nd	nd	nd	nd	nd	nd	MS, LRI, AD
Terpinen-4-ol	1182	1177	1.8 ± 0.6	3.8 ± 0.3	11.5 ± 0.6	nd	nd	nd	nd	nd	nd	MS, LRI, AD
*α*-Terpineol	1196	1188	nd	0.8 ± 0.1	nd	nd	nd	nd	nd	nd	nd	MS, LRI, AD
Myrtenal	1201	1195	nd	nd	2.3 ± 0.6	nd	nd	nd	nd	nd	nd	MS, LRI, AD
Verbenone	1213	1205	nd	nd	0.4 ± 0.1	nd	nd	nd	nd	nd	nd	MS, LRI, AD
Piperitone	1248	1252	nd	nd	0.2 ± 0.02	nd	nd	nd	nd	nd	nd	MS, LRI, AD
Isobornyl acetate	1289	1285	0.2 ± 0.1	0.5 ± 0.1	nd	nd	nd	nd	nd	nd	nd	MS, LRI, AD
Indole	1290	1291	nd	nd	nd	nd	nd	1.7 ± 0.9	nd	nd	nd	MS, LRI, AD
Bornyl acetate	1301	1288	nd	nd	0.6 ± 0.1	nd	nd	nd	nd	nd	nd	MS, LRI, AD
(*E*)-Pinocarvyl acetate	1303	1298	nd	nd	0.5 ± 0.2	nd	nd	nd	nd	nd	nd	MS, LRI, AD
*δ*-Elemene	1340	1338	0.3 ± 0.1	nd	nd	nd	nd	nd	nd	nd	nd	MS, LRI, AD
*α*-Cubebene	1353	1348	5.3 ± 0.6	nd	0.6 ± 0.1	nd	nd	nd	0.1 ± 0.06	nd	nd	MS, LRI, AD
Cyclosativene	1370	1371	nd	2.3 ± 0.4	2.8 ± 0.6	nd	nd	nd	nd	nd	nd	MS, LRI, AD
*α*-Ylangene	1374	1375	3.9 ± 0.8	nd	nd	0.7 ± 0.1	nd	nd	nd	nd	nd	MS, LRI, AD
*α*-Copaene	1380	1376	0.2 ± 0.1	1.3 ± 0.3	2.0 ± 0.5	nd	nd	nd	3.2 ± 0.6	nd	nd	MS, LRI, AD
*β*-Cubebene	1388	1388	0.89 ± 0.15	nd	nd	nd	nd	nd	nd	nd	nd	MS, LRI, AD
*β*-Elemene	1397	1390	nd	1.7 ± 0.3	3.0 ± 0.6	0.5 ± 0.1	nd	nd	3.8 ± 0.4	12.2 ± 1.3	3.6 ± 0.6	MS, LRI, AD
Longifolene	1413	1407	3.6 ± 0.6	nd	nd	nd	nd	nd	nd	nd	nd	MS, LRI, AD
(*E*)-Caryophyllene	1424	1419	nd	4.2 ± 0.4	3.2 ± 0.3	58.1 ± 1.3	94.8 ± 0.9	74.2 ± 1.5	21.2 ± 0.8	24.2 ± 1.6	6.0 ± 0.8	MS, LRI, AD
*β*-Copaene	1432	1432	0.6 ± 0.3	nd	0.2 ± 0.1	nd	nd	nd	nd	nd	nd	MS, LRI, AD
*γ*-Elemene	1437	1436	0.2 ± 0.1	nd	0.4 ± 0.1	nd	nd	nd	nd	nd	nd	MS, LRI, AD
*α*-Humulene	1456	1454	nd	nd	0.6 ± 0.1	0.8 ± 0.3	2.3 ± 0.5	2.0 ± 0.03	0.4 ± 0.05	1.4 ± 0.7	nd	MS, LRI, AD
(*E*)-*β*-Farnesene	1460	1456	nd	nd	nd	nd	nd	nd	0.1 ± 0.05	nd	nd	MS, LRI, AD
9-epi-(*E*)-Caryophyllene	1463	1466	0.5 ± 0.1	nd	nd	nd	nd	nd	0.5 ± 0.5	nd	nd	MS, LRI, AD
(*E*)-Cadina-1(6),4-diene	1474	1476	nd	1.3 ± 0.7	3.0 ± 0.4	nd	nd	nd	nd	nd	nd	MS, LRI, AD
*γ*-Muurolene	1480	1479	0.8 ± 0.2	nd	nd	nd	nd	nd	0.2 ± 0.1	nd	nd	MS, LRI, AD
*α*-Curcumene	1488	1480	nd	nd	nd	nd	nd	nd	3.5 ± 0.7	4.2 ± 0.4	2.9 ± 0.4	MS, LRI, AD
Germacrene D	1484	1485	0.2 ± 0.1	nd	nd	nd	nd	0.4 ± 0.1	nd	nd	nd	MS, LRI, AD
*δ*-Selinene	1486	1492	0.2 ± 0.1	nd	nd	nd	nd	nd	nd	nd	nd	MS, LRI, AD
(*Z*)-*β*-Guaiene	1489	1493	0.3 ± 0.1	nd	nd	nd	nd	nd	nd	nd	nd	MS, LRI, AD
*α*-Zingiberene	1501	1493	nd	nd	2.8 ± 0.3	0.2 ± 0.1	nd	nd	4.4 ± 0.7	11.7 ± 1.0	3.8 ± 0.5	MS, LRI, AD
Valencene	1496	1496	0.3 ± 0.1	nd	nd	nd	nd	nd	nd	21.2 ± 1.3	nd	MS, LRI, AD
Isodaucene	1497	1500	nd	4.1 ± 0.4	2.7 ± 0.4	nd	nd	nd	nd	nd	4.2 ± 1.0	MS, LRI, AD
*α*-Muurolene	1504	1500	0.6 ± 0.1	nd	nd	nd	nd	nd	nd	nd	nd	MS, LRI, AD
*β*-Bisabolene	1514	1505	nd	nd	nd	0.5 ± 0.1	nd	nd	2.7 ± 0.6	nd	nd	MS, LRI, AD
7-epi-*α*-Selinene	1520	1522	0.6 ± 0.1	nd	nd	nd	nd	nd	nd	nd	nd	MS, LRI, AD
*β*-Sesquiphellandrene	1527	1522	nd	nd	nd	nd	nd	nd	3.2 ± 0.5	4.8 ± 0.3	1.6 ± 0.6	MS, LRI, AD
*δ*-Cadinene	1529	1523	0.2 ± 0.1	nd	1.5 ± 0.4	0.3 ± 0.1	nd	0.4 ± 0.1	nd	nd	nd	MS, LRI, AD
(*E*)-*γ*-Bisabolene	1535	1531	nd	nd	nd	nd	nd	nd	0.63 ± 0.76	nd	nd	MS, LRI, AD
Germacrene B	1559	1561	nd	1.9 ± 0.9	4.2 ± 0.6	nd	nd	nd	nd	nd	nd	MS, LRI, AD
(*E*)-Nerolidol	1568	1563	0.2 ± 0.1	nd	1.8 ± 0.6	nd	nd	nd	nd	1.9 ± 0.8	nd	MS, LRI, AD
Caryophyllene oxide	1586	1583	nd	nd	4.4 ± 0.7	nd	nd	0.8 ± 0.1	nd	nd	1.9 ± 1.3	MS, LRI, AD
*γ*-Eudesmol	1627	1632	nd	nd	2.1 ± 0.5	nd	nd	nd	nd	nd	nd	MS, LRI, AD
*α*-Eudesmol	1648	-	nd	nd	4.1 ± 0.4	nd	nd	nd	nd	nd	nd	LRI, AD
Cryptomeridiol	1809	1813	nd	nd	5.0 ± 0.7	nd	nd	nd	nd	nd	nd	MS, LRI, AD
Cyclohexadecanolide	1936	1933	nd	nd	nd	nd	nd	nd	nd	nd	nd	MS, LRI, AD
Sandaracopimaradiene	1948	-	nd	nd	0.2 ± 0.1	nd	nd	nd	nd	nd	nd	LRI, AD
*n*-Hexadecanoic acid	1960	1959	nd	nd	8.7 ± 0.9	nd	nd	14.9 ± 0.4	nd	nd	55.7 ± 1.3	MS, LRI, AD
Number of constituents			37	19	36	16	2	9	20	13	14	
% of constituents identified			95.5%	96.9%	90.8%	99.1%	97.2%	97.1%	95.4%	91.8%	93.1%	
Yield (*v*/*w*)				0.4%	0.6%		0.2%	0.3%		0.2%	0.3%	
Monoterpene hydrocarbons			75.0%	74.3%	20.1%	38.0%	-	0.6%	51.5%	6.2%	-	
Oxygenated monoterpenes			2.0%	5.9%	17.6%	-	-	1.1%	-	4.0%	3.3%	
Sesquiterpene hydrocarbons			18.4%	16.7%	26.9%	61.1%	97.2%	77.0%	43.9%	79.7%	22.1%	
Oxygenated sesquiterpenes			0.2%	-	17.3%	-	-	1.7%	-	1.9%	1.9%	
Diterpene hydrocarbons			-	-	0.2%	-	-	-	-	-	-	
Fatty acid esters			-	-	8.7%	-	-	14.9%	-	-	55.7%	
Other compounds			-	-	-	-	-	1.7%	-	-	-	

Note: LRI Lit.: retention indices from literature by Adams [37]; LRI Cal.: experimentally determined; MS: identification by mass spectral database match with National Institute of Standards and Technology (NIST) and Wiley; LRI: linear retention index using the HP-5ms column (experimentally determined using the C_8_–C_20_ *n*-alkanes standard); AD: Adams database match [37]; nd: not detected; HD: hydrodistillation; OS: organic solvent; SPEM: solid-phase microextraction. Values are mean ± standard deviation (SD), *n* = 3.

**Table 2:** Total phenolic content, antioxidant activity, and tyrosinase inhibitory activity of *Z. kerrii* rhizome extract.

Sample	Total Phenolic Content (mg GAE/100 g Extract)	Antioxidant (IC_50_, μg/mL)		FRAP (mM AAE/g Extract)	Tyrosinase Inhibitory Activity (mg KAE/g Extract)
		DPPH	ABTS		
*Z. kerrii*	2.2 ± 0.1	143.6 ± 2.3	169.7 ± 41.0	0.3 ± 0.01	22.7 ± 0.8
Ascorbic acid	-	1.6 ± 0.8	5.2 ± 0.8	-	-

Note: GAE: gallic acid equivalence; AAE: ascorbic acid equivalence; KAE: kojic acid equivalence; DPPH: 2,2-diphenyl-1-picrylhydrazyl; ABTS: 2,2′-azino-bis(3-ethylbenzothiazoline-6-sulfonic acid) diammonium salt; FRAP: ferric ion reducing antioxidant power. Values are the mean ± SD, *n* = 3.

**Table 3:** Cytotoxicity of *Z. kerrii* rhizome extract on HaCaT cells after 24 h treatment.

Concentration (µg/mL)	% Cell Viability
12.5	98.7 ± 6.0
25	95.9 ± 6.9
s50	99.9 ± 0.3
100	97.5 ± 16.0
200	23.1 ± 10.4

Note: values are the mean ± SD, *n* = 3.

**Table 4:** Antibacterial activity of *Z. kerrii* rhizome extract.

Sample	Gram (+) Bacteria				Gram (−) Bacteria			
	*B. cereus*	*B. subtilis*	*S. aureus*	*S. epidermidis*	*E. coli*	*S. typhimurium*	*Ps. aeruginosa*	*Serratia marcescens*
*Z. kerrii*	1280	1280	640	1280	-	640	1280	-
Vancomycin	320	160	10	1280	-	-	-	-
Gentamycin	-	-	-	-	160	80	640	160
Ampicillin	-	320	5	320	80	640	1280	160
DMSO	1280	1280	-	1280	1280	-	1280	-

## References

[1] Sukari M.A., Sharif N.W.M., Yap A.L.C., Tang S.W., Neoh B.K., Rahmani M., Ee G.C.L., Taufiq-Yap Y.H., Yusof U.K. (2008). Chemical constituents variations of essential oils from rhizomes of four Zingiberaceae species. Malays. J. Anal. Sci..

[2] Thongam B., Sarangthem N., Konsam B. (2013). *Zingiber kerrii* (Zingiberaceae): A new record for India from Manipur. Taiwania.

[3] Triboun P., Chantaranothai P., Larsen K. (2007). Taxonomic changes regarding three species of *Zingiber* (Zingiberaceae) from Thailand. Acta Phytotax. Sin..

[4] Busatta C., Barbosa J., Cardoso R.I., Paroul N., Rodrigues M., Oliveira D., Oliveira J.V., Cansian R.L. (2017). Chemical profiles of essential oils of marjoram (*Origanum majorana*) and oregano (*Origanum vulgare*) obtained by hydrodistillation and supercritical CO_2_. J. Essent. Oil Res..

[5] Moradi M., Kaykhaii M., Ghiasvand A.R., Shadabi S., Salehinia A. (2011). Comparison of headspace solid-phase microextraction, headspace single-drop microextraction and hydrodistillation for chemical screening of volatiles in *Myrtus communis* L.. Phytochem. Anal..

[6] Pripdeevech P., Moonggoot S., Popluechai S., Chukeatirote E. (2014). Analysis of volatile constituents of fermented tea with *Bacillus subtilis* by SPME-GC-MS. Chiang Mai J. Sci..

[7] Pripdeevech P., Rothwell J., D’Souza P.E., Panuwet P. (2017). Differentiation of volatile profiles of Thai Oolong tea No. 12 provenances by SPME-GC-MS combined with principal component analysis. Int. J. Food Prop..

[8] Abbasi H., Rezaei K., Rashidi L. (2008). Extraction of essential oils from the seeds of pomegranate using organic solvents and supercritical CO_2_. J. Am. Oil Chem. Soc..

[9] Rehman S.U., Latief R., Bhat K.A., Khuroo M.A., Shawl A.S., Chandra S. (2017). Comparative analysis of the aroma chemicals of *Melissa officinalis* using hydrodistillation and HS-SPME techniques. Arab. J. Chem..

[10] Mesomo M.C., Corazza M.L., Ndiaye P.M., Santa O.R.D., Cardozo L., Scheer A.D.P. (2013). Supercritical CO_2_ extracts and essential oil of ginger (*Zingiber officinale* R.): Chemical composition and antibacterial activity. J. Supercrit. Fluids.

[11] Sivasothy Y., Chong W.K., Hamid A., Eldeen I.M., Sulaiman S.F., Awang K. (2011). Essential oils of *Zingiber officinale* var. rubrum Theilade and their antibacterial activities. Food Chem..

[12] Rana V.S., Ahluwalia V., Shakil N.A., Prasad L. (2017). Essential oil composition, antifungal, and seedling growth inhibitory effects of zerumbone from *Zingiber zerumbet* Smith. J. Essent. Oil Res..

[13] Bhuiyan M.N.I., Chowdhury J.U., Begum J. (2008). Volatile constituents of essential oils isolated from leaf and rhizome of *Zingiber cassumunar* Roxb. Bangladesh J. Pharmacol..

[14] Onyenekwe P.C., Hashimoto S. (1999). The composition of the essential oil of dried Nigerian ginger (*Zingiber officinale* Roscoe). Eur. Food Res. Technol..

[15] Danciu C., Vlaia L., Fetea F., Hancianu M., Coricovac D.E., Ciurlea S.A., Şoica C.M., Marincu I., Vlaia V., Dehelean C.A. (2015). Evaluation of phenolic profile, antioxidant and anticancer potential of two main representants of Zingiberaceae family against B164A5 murine melanoma cells. Biol. Res..

[16] El-Ghorab A.H., Nauman M., Anjum F.M., Hussain S., Nadeem M. (2010). A comparative study on chemical composition and antioxidant activity of ginger (*Zingiber officinale*) and cumin (*Cuminum cyminum*). J. Agric. Food Chem..

[17] Nag A., Bandyopadhyay M., Mukherjee A. (2013). Antioxidant activities and cytotoxicity of *Zingiber zerumbet* (L.) Smith rhizome. J. Pharmacogn. Phytochem..

[18] Koga A.Y., Beltrame F.L., Pereira A.V. (2016). Several aspects of *Zingiber zerumbet*: A review. Rev. Bras. Farmacogn..

[19] Chirangini P., Sharma G.J. (2005). In vitro propagation and microrhizome induction in *Zingiber cassumunar* (Roxb.) an antioxidant-rich medicinal plant. J. Food Agric. Environ..

[20] Masuda T., Jitoe A., Mabry T.J. (1995). Isolation and structure determination of cassumunarins A, B, and C: New anti-inflammatory antioxidants from a tropical ginger, *Zingiber cassumunar*. J. Am. Oil Chem. Soc..

[21] Bua-in S., Paisooksantivatana Y. (2009). Essential oil and antioxidant activity of Cassumunar ginger (Zingiberaceae: *Zingiber montanum* (Koenig) Link ex Dietr.) collected from various parts of Thailand. Kasetsart J..

[22] Manochaia B., Paisooksantivatanaa Y., Choib H., Hong J.H. (2010). Variation in DPPH scavenging activity and major volatile oil components of *Cassumunar ginger*, *Zingiber montanum* (Koenig), in response to water deficit and light intensity. Sci. Hortic..

[23] Phu N.D., Thy L.H.P., Lam T.D., Yen V.H., Lan N.T.N. (2019). Extraction of jasmin essential oil by hydrodistillation method and applications on formulation of natural facial cleansers. IOP Conf. Ser. Mater. Sci. Eng..

[24] Berker K.I., Olgun F.A.O., Ozyurt D., Demirata B., Apak R. (2013). Modified folin−ciocalteu antioxidant capacity assay for measuring lipophilic antioxidants. J. Agric. Food Chem..

[25] Li L.J., Li T.X., Kong Y.L., Yang M.H. (2016). Antioxidant aromatic butenolides from an insect-associated *Aspergillus iizukae*. Phytochem. Lett..

[26] Kanlayavattanakul M., Lourith N. (2011). Sapodilla seed coat as a multifunctional ingredient for cosmetic applications. Process Biochem..

[27] Li H.B., Wong C.C., Cheng K.W., Chen F. (2008). Antioxidant properties in vitro and total phenolic contents in methanol extracts from medicinal plants. LWT Food Sci. Technol..

[28] Abe S., Hirose S., Nishitani M., Yoshida I., Tsukayama M., Tsuji A., Yuasa K. (2018). Citrus peel polymethoxyflavones, sudachitin and nobiletin, induce distinct cellular responses in human keratinocyte HaCaT cells. Biosci. Biotechnol. Biochem..

[29] Septisetyani E.P., Ningrum R.A., Romadhani Y., Wisnuwardhani P.H., Santoso A. (2014). Optimization of sodium dodecyl sulphate as a formazan solvent and comparison of 3-(4,-5- dimethylthiazo-2-yl)-2,5-diphenyltetrazolium bromide (MTT) assay with wst-1 assay in mcf-7 cells. Indonesian J. Pharm..

[30] Zanette C., Pelin M., Crosera M., Adami G., Bovenzi M., Larese F.F., Florio C. (2011). Silver nanoparticles exert a long-lasting antiproliferative effect on human keratinocyte HaCaT cell line. Toxicol. In Vitro.

[31] Wikaningtyas P., Sukandar E.Y. (2016). The antibacterial activity of selected plants towards resistant bacteria isolated from clinical specimens. Asian Pac. J. Trop. Biomed..

[32] Masuda T., Yamashita D., Tadeda Y., Yonemori S. (2005). Screening for tyrosinase inhibitors among extracts of seashore plants and identification of potent inhibitors from *Garcinia subelliptica*. Biosci. Biotechnol. Biochem..

[33] Yoshimoto T., Yamamoto K., Tsuru D. (1985). Extracellular tyrosinase from *Streptomyces* sp. *KY*-453: Purification and some enzymatic properties. J. Biochem..

[34] Sasidharan I., Venugopal V.V., Menon A.N. (2012). Essential oil composition of two unique ginger (*Zingiber officinale* Roscoe) cultivars from Sikkim. Nat. Prod. Res..

[35] Singh G., Kapoor I.P.S., Singh P., Heluani C.S., Lampasona M.P., Catalan C.A.N. (2008). Chemistry, antioxidant and antimicrobial investigations on essential oil and oleoresins of *Zingiber officinale*. Food Chem. Toxicol..

[36] Ming J.C., Vera R., Chalchat J.C. (2001). Chemical composition of the essential oil from rhizomes, leaves and flowers of *Zingiber zerumber* Smith form Reunion Island. J. Essent. Oil Res..

[37] Adams R.P. (2009). Identification of Essential Oil Components by Gas Chromatography/Mass Spectrometry.

[38] Minatel I.O., Borges C.V., Ferreira M.I., Gomez H.A.G., Chen C.Y.O., Lima G.P.P. (2017). Phenolic compounds: Function properties, impact of processing and bioavailability. Phenolic Compounds—Biological Activity.

[39] Chan E.W.C., Lim Y.Y., Wong L.F., Lianto F.S., Wong S.K., Lim K.K., Joe C.E., Lim T.Y. (2008). Antioxidant and tyrosinase inhibition properties of leaves and rhizomes of ginger species. Food Chem..

[40] Hasan H.A., Raauf A.M.R., Razik B.M.A., Hassan B.A.R. (2012). Chemical composition and antimicrobial activity of the crude extracts isolated from *Zingiber officinale* by different solvents. Pharm. Anal. Acta.

[41] Chang L.W., Juang L.J., Wang B.S., Wang M.Y., Tai H.M., Hung W.J., Chen Y.J., Huang M.H. (2011). Antioxidant and antityrosinase activity of mulberry (*Morus alba* L.) twigs and root bark. Food Chem. Toxicol..

[42] Kim Y.J., Kang K.S., Yokozawa T. (2008). The anti-melanogenic effect of pycnogenol by its anti-oxidative actions. Food Chem. Toxicol..

[43] Prasad K.N., Yang B., Yang S., Chen Y., Zhao M., Ashraf M., Jiang Y. (2009). Identification of phenolic compounds and appraisal of antioxidant and antityrosinase activities from litchi (*Litchi sinensis* Sonn.) seeds. Food Chem..

